# Pathogenicity and Molecular Characterization of *Staphylococcus aureus* Strains Isolated from the Hospital Environment of CHU-Z Abomey-Calavi/Sô-Ava (Benin)

**DOI:** 10.1155/2021/6637617

**Published:** 2021-08-05

**Authors:** Akim Socohou, Haziz Sina, Cyriaque Degbey, Tomabu Adjobimey, Edna Sossou, Bawa Boya, Christine N'tcha, Hubert Adoukonou-Sagbadja, Adolphe Adjanohoun, Lamine Baba-Moussa

**Affiliations:** ^1^Laboratory of Biology and Molecular Typing in Microbiology, Department of Biochemistry and Cell Biology, Faculty of Sciences and Techniques, University of Abomey-Calavi, 05 BP 1604 Cotonou, Benin; ^2^Regional Institute of Public Health, University of Abomey-Calavi, Benin; BP 384 Ouidah, Benin; ^3^Biochemistry and Molecular Biology Unit, Department of Biochemistry and Cellular Biology, Faculty of Sciences and Techniques, University of Abomey-Calavi, 05 BP 1604 Cotonou, Benin; ^4^Department of Genetics and Biotechnology, Faculty of Science and Technology (FAST), University of Abomey-Calavi (UAC), BP, 1947 Cotonou, Benin; ^5^National Institute of Agronomic Research of Benin, Benin

## Abstract

*Staphylococcus aureus* is a major human pathogen present on a third of the healthy population. The bacterium possesses an extensive arsenal of virulence factors. The pathogenicity is linked with *S. aureus* high plasticity and its exceptional ability to incorporate foreign genetic material. The aim of the present study was to perform molecular characterization of *Staphylococcus aureus* strains isolated from the clinical environment of the CHU-Z Abomey-Calavi/Sô-Ava. Isolation of *Staphylococcus aureus* bacterium was performed on Chapman agar. Toxin production by isolated *S. aureus* strains was investigated using the radial immunoprecipitation technique. A colorimetric assay was used to evaluate *Staphylococcus aureus* lipase (SA-Lipase) production. Finally, the expression of antibiotic resistance genes and genes encoding toxins production was investigated. Our data suggest that none of the isolated *Staphylococcus aureus* strains expressed the investigated toxin genes. Interestingly, SA-Lipase was produced by 14.28% of our isolated *S. aureus* strains. The *mec*A gene was present in 57.14% of the isolated strains, while PVL and TSST-1 genes were identified in 2.85 and 7.14% of *S. aureus,* respectively. Significant genetic diversity was observed along the hospital environment *S. aureus* strains. The present study reveals the level of virulence of *S. aureus* strains isolated in the different units of CHU-Z Abomey Calavi/Sô-Ava through the production of lipase, PVL, and epidermolysins. The molecular study has favored a genetic characterization within the isolated strains.

## 1. Introduction

*Staphylococcus aureus* bacterium is often encountered in human pathology and is the causative agent of a wide variety of infections including life-threatening toxic shock syndrome, pneumonia, and sepsis. The clinical outcome of these infections depends on numerous virulence factors that *S. aureus* can produce. Staphylococcal infections represent a major public health problem both because of the bacteria's virulence and the emergence of multiresistant strains [1]. Thus, *S. aureus* infections are challenging both in terms of diagnosis and therapeutic strategies [2]. Furthermore, *Staphylococcus* resistance to methicillin is mainly attributed to the acquired *mec* A gene [3]. In addition, a new mecC gene has recently emerged especially in *S. aureus* of animal origin [4]. Several studies show that multidrug-resistant *S. aureus* strains are involved in infections and hospital-related deaths in both industrialized and developing countries [5].

In addition, the pathogenicity of this bacterium is linked to the expression of virulence factors [6]. Indeed, *S. aureus* can produce various heat-resistant extracellular protein toxins that behave as virulence factors, including superantigens, hemolysins, and leukocidins. The most important of these factors is the Panton-Valentine leukocidin (PVL), which is a cytotoxin that forms pores in the membrane and has been associated with boils, skin abscesses, and severe necrotic skin infections [7, 8]. Some of the pathogenic *S. aureus* strains also possess the gene coding for staphylococcal toxic shock toxin (TSST-1) [9]. These toxins have superantigenic activities and are capable of polyclonal T-cell activation independently of their antigenic specificity. This activation is directly linked to the clinical signs of staphylococcal toxic shock [10, 11]. It has been shown that the secretion of these toxins by strains in infections constitutes a risk of prolonged hospitalization periods [12].

*S. aureus* can produce abundant amounts of secreted glycerol ester hydrolase (lipase) enzymes, which release free fatty acids from triglycerides [13]. Indeed, some triglycerides contain additional toxic fatty acids that appear to interfere with cell growth by altering cell permeability, cutting off oxidative phosphorylation, or blocking electron transport [14]. This broad array of pathogenic profiles is linked to the high genomic plasticity of *S. aureus*, which confers to the bacteria an exceptional ability to incorporate exogenic genetic material from other strains and thus to acquire new properties, including antibiotic resistance and virulence [13]. All these factors make *S. aureus* a predominant nosocomial pathogen among immune-compromised patients in hospital environments and health care facilities [15]. The present study is aimed at evaluating the genetic diversity and virulence capacity of *S. aureus* strains isolated from the hospital environment of CHU-Z Abomey-Calavi/Sô-Ava.

## 2. Material and Methods

### 2.1. DNA Extraction

A total of 42 S*. aureus* isolates previously isolates from the hospital environment of CHU-Z Abomey-Calavi/Sô-Ava in Benin [16] were used in this study. Those isolates were collected from five medical units of CHU-Z Abomey-Calavi/Sô-Ava: pediatrics (10 isolates), operating room (7 isolates), neonatology (10 isolates), maternity (8 isolates), and central sterilization room (7 isolates). Purification of isolates DNA was done as previously described by Rasmussen and Morrissey [17]. Briefly, 1.5 ml microcentrifuge tubes containing bacterial strains were centrifuged at 12000 rpm for 5 min, the supernatant was poured, and 500 *μ*l of sterile distilled water was added to the bacterial pellet. The mixture was then heated in a dry bath at 95°C for 15 min. The tubes were centrifuged again at 12000 rpm for 5 min, and the obtained supernatants were recovered in new tubes. 500 *μ*l of absolute ethanol was added, and the tubes were centrifuged at 12000 rpm for 5 min. The DNA pellets were suspended in 50 *μ*l sterile distilled water and maintained at 4°C for immediate use or at -20°C for long-term storage.

### 2.2. Molecular Identification of *S. aureus* by the 16S-23S rRNA Gene

The 16S-23S rRNA gene primers [18] were used for the molecular confirmation of the *S. aureus* isolates. The sequences of the primers are *G*1 (5′-GAAGTCGTAACAAGG-3′) and *L*1 (5′-CAAGGCATCCACCGT). The PCR reaction was performed on 25 *μ*l containing 20 *μ*l 10x GoTaq mix (PROMEGA, Madison, WI USA), 1 *μ*l primer *G*1 (10 *μ*M), 1 *μ*l primer *L*1 (10 *μ*M), and 3 *μ*l DNA. The amplification program is composed of an initial denaturation (94°C for 2 min), 30 cycles of denaturation cycles (94°C, 1 min), hybridization (50°C, 2 min), elongation (72°C, 2 min), and a final elongation (72°C, 7 min). For the classification base on the results, 2 patterns were considered different if 2 or more bands of the electropherogram differed in size.

### 2.3. Production of Leukotoxins and Epidermolysins

The production of Panton and Valentine Leukotoxin (PVL), LukE-LukD leukotoxin, and epidermolysins (ETA, ETB) of *S. aureus* isolates was investigated by radial immunoprecipitation [19]. Briefly, fresh *S. aureus* colonies were cultured in 500 *μ*l of Yeast Casamino-acid Pyruvate (YCP) broth in 12-well culture plates and incubated at 37°C for 18-24 hours under agitation (200 rpm). The content of each well was poured into microcentrifuge tubes, and culture supernatants were collected (using a micropipette) after centrifugation at 5,000 rpm for 5 min. On a 0.6% agarose gel, 7 wells spaced 8 mm were dug in a rosette. Approximately 30 *μ*l of each sample is placed in the corresponding outer well, followed by purified rabbit anti-leukotoxin (PVL and LukE-LukD) and antiepidermolysins (ETA and ETB) antibodies (OD = 3), placed in the rosette's central well as control antigens (OD = 0.2) in the upper and lower wells in a clockwise direction. After a 16-hour diffusion, the precipitation arcs were viewed directly with the naked eye or after staining with Coomassie blue [19].

### 2.4. Lipase Production Capability

The procedure described by Janda [20] was used to detect the production of lipolytic enzymes in which polyoxyethylene sorbitan monooleate (Tween 80) is used as a lipid substrate. This method is based on the precipitation of the calcium crystals salt in presence of fatty acid induced by lipase. If the bacterial strain has a lipolytic enzyme, the calcium soap crystals' precipitation will be visible as an opaque halo around the colonies. In addition, if the lipase activity is intense, the precipitation will appear as crystals visible to the naked eye.

The bacterial strains are therefore inoculated on nutrient agar supplemented with Tween 80. The substrate is added to the culture medium, previously sterilized, at a concentration of 1%. The appearance of opaque precipitation around bacterial colonies occurs after 24 to 72 hours of incubation at 37°C.

### 2.5. Search of mecA Gene

The primers used to target the mecA gene were those designed by Malik et al. [21]. The primers sequences are mecA F: 5′- TCCAGGAATGCAGAAAGACC-3′ and mecA R: 5′-TCACCTGTTTGAGGGTGGAT-3′. The 25 *μ*l reaction mixture contained 20 *μ*l GoTaq mix 10x (PROMEGA, Madison, WI USA), 1 *μ*l primer F (10 *μ*M), 1 *μ*l primer R (10 *μ*M), and 3 *μ*l DNA.

The amplification program is initial denaturation (94°C, 3 min), 35 cycles of denaturation (94°C, 1 min), hybridization (55°C, 1 min), elongation (72°C, 1 min), and a final elongation (72°C, 10 min). The expected fragment size of the PCR product is 675 bp.

### 2.6. Detection of Genes Encoding the Production of Toxins

In order to confirm certain immunological results, the presence of genes encoding Panton and Valentine Leukotoxin (PVL: F: 5′-AAATGCCACTGTTATCCAGAGGTA-3′ and R: 5′-T TTGCAGCGTTTTGTTTTCG-3′) and Toxic Shock Syndrome Toxin 1 (TSST-1: F: 5′- ACCCCTGCCTTTCCATCATC-3′ and R: 5′-T TTTCAGTATTTGTAACGCC-3′) was investigated using specific primers. The 25 *μ*l reaction mixture contained 18 *μ*l GoTaq mix 10x (PROMEGA, Madison, WI USA), 1 *μ*l primer (LPV) F (10 *μ*M), 1 *μ*l primer (LPV) R (10 *μ*M), 1 *μ*l primer (TSST-1) F (10 *μ*M), 1 *μ*l primer (TSST-1) R (10 *μ*M), and 3 *μ*l DNA. The toxin amplification program was initial denaturation (92°C, 2 min), 35 cycles of denaturation (92°C, 2 min), hybridization (50°C, 1 min), elongation (72°C, 2 min), and a final elongation (72°C, 3 min).

### 2.7. Data Analysis and Processing

The data collected was coded and processed using Microsoft Excel 2013 spreadsheet software. Graph Pad Prism 7 software was used for comparison testing of positive isolates at various collection sites. R software was used for correspondence factor analysis and then using the results of this analysis for dendrogram clustering. Significance was accepted when *p* < 0.05.

## 3. Results

### 3.1. Genotypic Characterization of *S. aureus* Strains

#### 3.1.1. Genotypic Identification of *S. aureus* by 16S-23S rRNA

**Figure 1:**
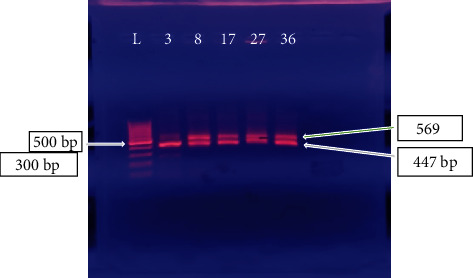
Photo showing the bands of the 16S-23S rRNA gene. L: Molecular weight marker; *S. aureus samples*: (3, 8, 17, 27, 36).

Observation of UV gels after amplification reveals the presence of several bands within isolated *S. aureus* strains. The number of bands varies from 2 to 4 bands in our study. Band sizes range from about 300 bp, about 350 bp, and about 447 to 569 bp (Figure 1). However, the predominant characteristic stamp was the presence of both bands (447 bp and 569 bp). This confirms that 35 strains out of 42 were effectively *Staphylococcus aureus*.

**Figure 2:**
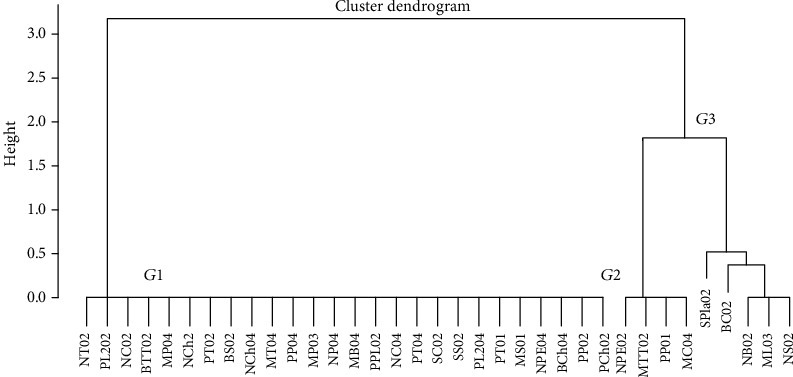
Genetic diversity dendrogram as a function of the 16S-23S rRNA gene. The initials indicate the service: N: neonatology; M: maternity; P: pediatrics; B: operating room; S: central sterilization.

Using the polymorphism based on the size and number of bands, the studied *S. aureus* isolates were classified. Thus, considering scale 1, three phylogenetic subgroups of *Staphylococcus aureus* were obtained (Figure 2). Notable was that the strains of *S. aureus* that make up subgroup 1 are related and originate from the five services studied. On the other hand, strains in subgroup 2 were from the neonatal, pediatric, and maternity wards. Furthermore, subgroup 3 comprises strains from four services (central sterilization, operating room, maternity, and neonatology).

### 3.2. Toxigenic Profile of Isolated *S. aureus* Strains

#### 3.2.1. Phenotypic Production of Toxins and Lipase

On reading the immunoprecipitation test, it appears that no strain of *S. aureus* produces PVL toxin, and the same observation was made for epidermolysin A (ETA) and epidermolysin B (ETB) (Table 1). On the other hand, the lipase test revealed that at least one strain in each of the departments such as pediatrics, neonatology, and central sterilization has produced lipase, but in the maternity ward, 2 strains were observed to produce lipase (Table 1). However, the strains of *S. aureus* isolated in the operating theatre did not produce lipase. In general, 14.28% of the *S. aureus* strains isolated in the hospital environment were lipase-producing.

#### 3.2.2. Detection of the mecA, LPV, and TSST-1 Gene

After PCR, the distribution of the presence of the mecA gene by service shows that bacterium isolated in neonatology are more carriers of the mecA resistance gene, followed by maternity and pediatrics (Table 2). On the other hand, it appears that only one strain of *S. aureus* has the Panton and Valentine leukotoxin (PVL) gene. This strain with the PVL gene is isolated in the maternity ward. In contrast, for the TSST-1 gene, three strains of *S. aureus* possess this gene and are from pediatrics, maternity, and central sterilization (Table 2).

**Figure 3:**
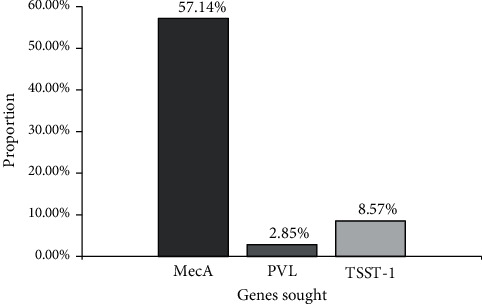
Proportion of genes searched.

Globally, it should be noted that 57.14% of our *S. aureus* strains have the methicillin resistance gene (mecA) in their genome. 2.85% and 8.57% represent the proportion expressing the PVL and Toxic Shock Toxin genes, respectively (Figure 3).

## 4. Discussion

The molecular technique showed large importance of its use since 7 strains were not *S. aureus*. The 16S-23S ribosomal RNA gene can be said to be a target of choice and useful for better strain identification [22]. The reason for the genetic polymorphism (band size and number of bands) linked to the 16S-23S rRNA gene observed in our study is due to the presence of multiple copies of this gene in *S. aureus* strains. Several authors have shown that *S. aureus* was the species with the highest number of polymorphisms [18, 23, 24]. Furthermore, the characteristic band profile (about 447 bp and 569 bp) is also observed in the work of Mendoza et al. [25] and Akineden et al. [26].

Concerning the pathogenicity of *S. aureus*, one of the strategies adopted by host cells to respond to infection is the production of fatty acids and lipids, which form small holes in the bacterial membrane. In contrast, *S. aureus* produces enzymes called lipases that destroy these fatty acids before causing damage to the bacterial membrane [27]. It appears that 14.28% of our *S. aureus* strains are lipase producers. This result could be explained by the low use of fats in the composition of detergents used in surface cleaning. Also, lipase production in the test with Tween 80 is justified by the stimulating effect of Ca^2+^ present in the medium in *S. aureus*. This is confirmed by Bornsheur et al. [28], who state that bivalent cations such as calcium often stimulate the enzymatic activity of lipase. Recent work by Cadieux et al. [29] reported lipase production by strains of *S. aureus*, *S. hycus* USA 300, and Pogaku et al. [30] in *Staphylococcus* sp. Lp12 strains. Therefore, this virulence factor deserves more attention because microbial lipases are highly stable to temperature, detergents, and certain proteolytic enzymes [31].

The phenotypic results on the nonproduction of PVL, ETA, and ETB toxins by our *S. aureus* strains are partly reassuring. The same observations were made by Ahoyo et al. [32] in *S. aureus* strains isolated in the hospital environment in central Benin and by Sina et al. [33] in *S. aureus* isolated from catheters at the CNHU of Cotonou. On the other hand, many clinical strains isolated from body fluids produce PVL up to more than 50% of the bacterium isolated [33, 34]. This difference could be due to the fact that strains isolated from body fluids are often confronted to the immune system and have developed this virulence factor. However, molecular PCR assays showed that only one strain had the PVL gene, and three strains of *S. aureus* had the TSST-1 gene. And the mere fact that these strains came from the pediatric and maternity wards is worrisome given the proximity of the two wards in the hospital and the risk of probable gene transfer. Our results on the presence of the PVL gene are different from the 7.14% obtained by Cadé et al. [35] in France. For the TSST-1 gene, the 7.14% obtained in our study is slightly higher than the 3.3% obtained by Baba-Moussa et al. [7] in *S. aureus* isolated from dermatoses.

This variation could be attributed to the level of pathogenicity of the strains or the geographical location but also to the difference in gene detection methods. Although the pathogenicity of these strains does not appear to be related to the production of PVL based on the immunological results obtained, it is easy to understand that the presence of the genes that can promote the expression of these different toxins is associated with an increase in virulence of certain strains of *S. aureus* [2]. Thus, the primary concern remains in the possibility of horizontal transfer of these genes and their possible expression within *S. aureus* species, which could promote the spread of virulent strains in the coming years. In addition, certain hereditary immune deficiencies are associated with severe PVL+ *staphylococcus* infections: chronic septic granulomatosis, leukocyte adhesion defects, congenital neutropenia, and innate immunity defects. However, these strains (PVL+) are known to be responsible, particularly in nonimmunocompromised children, for necrotizing pneumopathies, rapid progression, and poor prognosis despite appropriate antibiotic therapy [36].

However, methicillin resistance is also one of the criteria for virulence of *Staphylococcus aureus*. Indeed, the phenotypic expression of methicillin resistance is influenced by several parameters (mean temperature, pH, and NaCl content in the medium and even genotypic). Thus, the presence of the mecA gene by the molecular method gives a clearer idea of the MRSA strains in our samples. We deduce that at least half of the *S. aureus* strains isolated from neonatal, maternity, and pediatric wards are carriers of the mecA gene. The contiguous nature of these three departments would be a critical factor in bringing the three departments closer together, thus, favoring a probable transfer of the mecA gene and also the diffusion of its resistant bacterium through the movement of staff from one department to another or of patients from one department to another.

Overall, 57.14% of isolated *S. aureus* possess the mecA gene. These results are consistent with those of Rocchetti et al. [37], who reported that 50.6% of *S. aureus* isolated from blood culture bottles in Brazil had the mecA gene. On the other hand, our results are contrary to those of Sajit Khan et al. [38] in South India, who found that 94% of clinical MRSA were carriers of the mecA gene. This difference could be explained by a low transfer of the mecA gene within *S. aureus* strains from CHU-Z Abomey-Calavi/Sô-Ava. Furthermore, several studies show that the mecA gene is also present in coagulase-negative staphylococci [37]. It is important that this work can help improve new therapy options to limit the spread of these pathogens. Because hospital-acquired MRSA is also responsible for community-acquired infections in patients with risk factors such as a history of hospitalization [39].

## 5. Conclusion

This work allows to highlight the level of virulence of *S. aureus* strains isolated in the departments of CHU-Z Abomey-Calavi/Sô-Ava through the production of lipase as well as the production of PVL and epidermolysins. PCR research has shown the presence of the mecA gene and the PVL gene in our *S. aureus* strains. The molecular study favored a genetic characterization within our strains, which allowed a group matching. The acquisition of these virulence factors as well as the genetic background of these strains requires more attention and suggests the necessity of specific hospital measures.

## Figures and Tables

**Table 1:** Production of virulence factors by service.

Virulence factors	Services					
	Pediatrics	Maternity	Neonatology	Operating room	Central sterilization	Total
PVL	0	0	0	0	0	0/35
ETA	0	0	0	0	0	0/35
ETB	0	0	0	0	0	0/35
Lipase	1	2	1	0	1	5/35

**Table 2:** Result of the search for virulence genes.

Gene sought	Services					
	Pediatrics	Maternity	Neonatology	Operating room	Central sterilization	Total
MecA	5	5	7	2	1	20/35
PVL	0	1	0	0	0	1/35
TSST-1	1	1	0	0	1	3/35

## Data Availability

The data used to support the findings of this work are available from the corresponding author upon request.
